# Integrated widely targeted UPLC-MS/MS metabolomics and transcriptomics reveal MYB-linked variation in bioactive phenolics across five kiwifruit varieties

**DOI:** 10.1016/j.fochms.2026.100410

**Published:** 2026-05-09

**Authors:** Mingzheng Duan, Jieyu Chang, Shirong He, Yuanqiao Li, Congjing Chen, Tingfen Lu, Jinrui Duan, Xiande Duan, Qurban Ali, Shunqiang Yang, Muhammad Junaid Rao

**Affiliations:** a Advanced Institute of Ecological Agriculture and Biodiversity on the Yunnan-Guizhou Plateau/Yunnan Key Laboratory of Smart Villages and Agri-Cultural-Tourism Integration, Zhaotong University, Zhaotong 657000, China; b State Key Laboratory for Development and Utilization of Forest Food Resources, Zhejiang A&F University, Hangzhou 311300, China; c Department of Biology, College of Science, United Arab Emirates University, Al-Ain, Abu-Dhabi, United Arab Emirates

**Keywords:** Kiwifruit phenolic compounds, *Actinidia arguta*, Metabolomics, Radical-scavenging capacity, MYB genes

## Abstract

Kiwifruit (*Actinidia* spp.) is valued for its bioactive phytochemicals, yet the phenolic metabolites across species and varieties remain underexplored. Using widely targeted ultra-performance liquid chromatography coupled with tandem mass spectrometry (UPLC-MS/MS) metabolomics, we profiled phenolics in five varieties representing *Actinidia chinensis* (‘SunGold’, ‘Guichang’, wild) and *Actinidia arguta* (‘Danyang flat’ DY, ‘Maolvfeng’). A total of 190 phenolic metabolites were identified. The DY strongly accumulated lignans and coumarins, with cleomiscosin C (log₂FC 14.09 vs. ‘SunGold’), fraxin, schizandriside, etc., markedly up-regulated and exhibited the highest radical-scavenging capacity. Multivariate analyses confirmed distinct varietal clustering and identified Variable Importance in Projection (VIP) > 1.0 compounds driving separation. Species-specific MYB transcription factor expression correlated with these phenolic profiles, indicating a genetic-regulatory basis for the divergent accumulation. Pathway analysis revealed that genetic background diverts carbon into specific phenolic pathways: *A. arguta* preferentially produces lignans/coumarins, whereas ‘SunGold’ enriches flavonoids. These results provide a metabolic and transcriptional map to support targeted breeding for nutritionally enhanced kiwifruit varieties.

## Highlights

• UPLC-MS/MS identified 190 phenolic metabolites across five kiwifruit varieties.
• *arguta* ‘Danyang flat’ strongly accumulates lignans and coumarins.
• ‘SunGold’ exhibits a flavonoid-enriched phenolic profile.
• Highest radical-scavenging capacity correlates with total phenols in ‘Danyang flat’.
• MYB transcription factor expression links to species-specific phenolic accumulation.

## 1 Introduction

Kiwifruit (*Actinidia* spp.) is globally recognized as a nutrient-dense fruit valued for its distinctive flavor, vibrant colour, and exceptional nutritional and functional quality. Beyond its high vitamin C content, kiwifruit is a rich source of bioactive phenolic compounds that contribute to both its sensory quality and functional attributes (Zhang et al., 2020). Phenolics play a central role in determining fruit colour, astringency, and bitterness, while also imparting potent antioxidant, anti-inflammatory, and cardioprotective effects (Rao et al., 2025; Rao & Zheng, 2025). These attributes have positioned kiwifruit as a model fruit for exploring the interface between food composition, functionality, and nutritional quality (Moysidou et al., 2024). In the context of food chemistry, understanding the diversity and regulation of phenolic metabolites in kiwifruit is critical for optimizing its nutritional value and guiding breeding strategies toward enhanced functional quality.

Current research has identified flavonoids and phenolic acids as the predominant phenolic classes in kiwifruit, with compounds such as quercetin glycosides, catechins, and chlorogenic acid being frequently reported (Moysidou et al., 2024; Yu et al., 2020). These metabolites are integral to the fruit's antioxidant capacity and contribute to its characteristic sensory profile. However, the phenolic landscape of kiwifruit extends beyond these well-characterized compounds. Emerging evidence suggests the presence of other phenolic subclasses, including coumarins, lignans, and stilbenes, though their distribution and functional significance remain poorly understood (Li et al., 2024; Yu et al., 2020; Zhang et al., 2020). Moreover, most studies have focused on a limited number of varieties within *Actinidia chinensis* (yellow-fleshed) and *Actinidia deliciosa* (green-fleshed) species, while comparatively less is known about *A. arguta* (hardy kiwifruit), which exhibits distinct metabolic and sensory characteristics. The genetic diversity across these species likely underpins substantial variation in phenolic composition, yet comprehensive comparative metabolomic analyses remain scarce (Li et al., 2024). This knowledge gap limits our understanding of how species-specific metabolic pathways contribute to the nutritional and functional diversity of kiwifruit.

At the molecular level, the biosynthesis of phenolic compounds in plants is tightly regulated by transcription factors, among which the MYB family plays a pivotal role (Rao & Zheng, 2025). MYB transcription factors orchestrate the expression of structural genes in the phenylpropanoid pathway, influencing the accumulation of flavonoids, phenolic acids, and related metabolites (Rao, Duan et al., 2025; Rao & Zheng, 2025). Recent reviews highlight the central role of transcription factors in bridging plant stress responses and metabolic regulation (Liu et al., 2024). In several fruit crops, MYB regulators have been linked to variations in pigmentation, antioxidant potential, and stress responses (Duan et al., 2025). Despite the availability of genomic resources for kiwifruit, the functional characterization of MYB transcription factors in relation to phenolic biosynthesis remains limited. Preliminary transcriptomic studies have identified MYB candidates potentially associated with flavonoid and lignin pathways (Mao et al., 2024; X. Wang et al., 2021), yet the extent to which MYB expression patterns modulate phenolic diversity across kiwifruit species is largely unexplored. Elucidating these regulatory relationships is essential for establishing genetic-metabolic linkages that can inform targeted breeding for enhanced phenolic content and functionality.

The diversity of phenolic metabolites in kiwifruit is not merely of academic interest; it has direct implications for nutritional functionality, antioxidant capacity, and consumer appeal (Barzegar et al., 2025; Duan et al., 2026; Zhang et al., 2020). Phenolic compounds contribute to the fruit's ability to scavenge reactive oxygen species, modulate gut microbiota, and influence postharvest stability (Guo et al., 2026; Zhang et al., 2020). Recent comparative studies have demonstrated that pigmented kiwifruit cultivars, including the red-fleshed *A. chinensis* ‘Hongyang’ and the purple-fleshed *A. arguta* ‘Mini Amethyst’, exhibit markedly higher concentrations and diversity of flavonoids, highlighting their enhanced nutritional attributes (Yu et al., 2020). In addition, wild accessions frequently present unique chemotypic profiles characterized by elevated accumulations of flavan-3-ols, compounds that are closely associated with beneficial physiological functions (Asadi et al., 2024). From a food chemistry perspective, understanding the interplay between phenolic composition and antioxidant activity provides a foundation for designing functional foods and nutraceuticals with optimized functional outcomes. Importantly, this variation is orchestrated by the differential expression of pivotal biosynthetic genes (such as *F3H*, *UFGT* etc.) and associated transcription factors (such as bHLH and MYB10), which modulate the metabolic flux within phenolic biosynthetic pathways (Asadi et al., 2024). The phenolic profile thus serves as a biochemical marker for varietal differentiation and quality assessment, offering valuable insights for breeding programs aimed at improving both sensory and nutritional traits. In this context, integrating metabolomic and transcriptomic approaches offers a powerful strategy to unravel the complex regulatory networks governing phenolic biosynthesis and accumulation.

Despite growing interest, a comprehensive understanding of phenolic composition across diverse kiwifruit varieties remains incomplete. The current literature lacks systematic, comparative profiling of phenolic subclasses across diverse kiwifruit species and varieties. Moreover, the genetic basis underlying phenolic variation and their correlated MYB genes, has not been clearly established. This gap constrains the ability to exploit natural phenolic diversity for breeding nutritionally superior cultivars. Addressing this limitation requires an integrative approach that combines metabolomics with gene expression analysis to link metabolic phenotypes with regulatory mechanisms. The present study aims to fill this knowledge gap by systematically characterizing the phenolic metabolites of five kiwifruit varieties representing *A. chinensis* and *A. arguta* species using ultra-performance liquid chromatography coupled with tandem mass spectrometry (UPLC-MS/MS). Specifically, the objectives are to (i) comprehensively profile phenolic metabolites, including flavonoids, phenolic acids, coumarins, and lignans; (ii) compare phenolic accumulation patterns between *A. chinensis* and *A. arguta* to elucidate species-specific metabolic signatures; (iii) investigate the correlation between MYB gene expression and phenolic profiles to identify key regulatory associations; and (iv) determine the relationship between phenolic composition and radical-scavenging capacity to identify discriminatory metabolites contributing to functional quality. By integrating metabolomic and transcriptional analyses, this study seeks to provide a mechanistic understanding of phenolic diversity in kiwifruit and establish a molecular basis for nutrition-oriented breeding. The findings are expected to advance the field of food chemistry by linking compositional diversity to functional outcomes, thereby enhancing the scientific foundation for developing kiwifruit varieties with superior nutritional and functional quality.

## 2 Material and methods

### 2.1 Preparation of kiwifruit samples

We analyzed five kiwifruit varieties: LC (‘Maolvfeng’), WL (wild), GC (‘Guichang’), SG (‘SunGold’), and DY (‘Danyang flat’). The five varieties belong to two species: *Actinidia chinensis* (GC, WL, SG) and *Actinidia arguta* (LC, DY). Fruits of consistent ripeness and free from physical damage or pests were selected. All fruits underwent the same post-harvest handling. They were first rinsed gently with clean water to remove surface debris, then air-dried on sterile gauze. Using a sterile scalpel, the skin was carefully removed from each fruit, and only the flesh was retained. For each variety, flesh from several fruits was pooled and ground into a uniform pulp to form one biological replicate. Three independent biological replicates were prepared per variety.

A portion of the homogenized pulp was wrapped tightly in aluminum foil to form a small sphere, placed into pre-chilled centrifuge tubes, and labeled appropriately. The samples were flash-frozen by submerging the tubes in liquid nitrogen for 2–5 min until completely solid. Finally, all samples were stored in a − 80 °C freezer for subsequent metabolic profiling.

Pedoclimatic conditions and origin of varieties: GC and WL were collected from Weixin County, Zhaotong, Yunnan, China (altitude 1200–1600 m; annual average temperature 13–14 °C; annual precipitation 960–1100 mm; yellow-brown loam soil). LC and DY were obtained from Dandong, Liaoning, China (altitude ∼20 m; annual average temperature 8–10 °C; annual precipitation 900–1200 mm; brown forest soil/ brown loam soil). SG was sourced from a commercial orchard in New Zealand (temperate maritime climate; sandy loam soil; standard horticultural management). Voucher specimens for each variety were deposited at the Herbarium of Zhaotong University (accession numbers ZTU-2023-001 to ZTU-2023-005).

### 2.2 Chemicals and reagents

All chemicals and solvents were of analytical or HPLC grade. For the DPPH● radical scavenging assay, DPPH (1,1-diphenyl-2-picrylhydrazyl, CAS 1898-66-4) and the standard Trolox (6-hydroxy-2,5,7,8-tetramethylchroman-2-carboxylic acid) were used, with sample extraction performed in 80% (*v*/v) methanol. For the UPLC-MS/MS metabolomic analysis, HPLC-grade acetonitrile, methanol, and formic acid were supplied by Merck KGaA (Darmstadt, Germany). Ultra-pure water was produced using a Milli-Q water purification system (Millipore, Bedford, MA, USA). The internal standard, 2-chlorophenylalanine (98% purity, CAS 14091–11-3), was obtained from J&K Scientific Ltd. (Beijing, China). Metabolite extraction was carried out using 70% (v/v) methanol containing 1 ppm of this internal standard, and chromatographic separation employed mobile phases of water (A) and acetonitrile (B), each modified with 0.1% (v/v) formic acid.

### 2.3 DPPH● radical scavenging assay

DPPH● radical scavenging capacity of five kiwifruit varieties was determined using a commercial kit (G0128W, Grace Biotechnology Co., Ltd., Suzhou, China) according to the manufacturer’s microplate instructions. Fresh flesh samples (∼0.1 g) were homogenized in 1 mL 80% methanol and extracted by ultrasonication (60 °C, 200–300 W, 30 min) with intermittent vortexing, then centrifuged (12 000 rpm, 10 min). The supernatant, diluted if necessary, was reacted with DPPH working solution (150 µL extract + 150 µL ethanol for test, 150 µL extract + 150 µL DPPH for control, blank: 150 µL 80% methanol + 150 µL DPPH). After 30 min dark incubation at 25 °C and brief centrifugation, absorbance at 517 nm was read in a 96‑well plate. Clearance (%) was calculated as [1 − (A_test − A_control)/A_blank] × 100 and converted to Trolox equivalents via the standard curve (clearance = 2.8486 × [Trolox] + 0.7084). Results were expressed as µg Trolox/g fresh weight.

### 2.4 Widely targeted UPLC-MS/MS metabolomics of phenolic compounds

Polyphenol profiles across the five kiwifruit varieties were characterized using a widely targeted metabolomics approach on an UPLC-MS/MS system (Wuhan Metware Biotechnology Co., Ltd.). For each variety, three independent biological replicates were processed. Fresh fruit samples were freeze-dried and ground into a fine powder. From this, 30 mg of powder was extracted with 1500 μL of a chilled 70% methanol solution, which contained 2-chlorophenylalanine (1 ppm) as an internal standard for normalization. After vortexing and centrifugation, the supernatant was passed through a 0.22 μm filter for analysis. A pooled quality control (QC) sample was prepared by mixing 10 μL of every extracted sample (from all varieties and replicates), followed by vortexing and centrifugation. The QC sample was analyzed after every 6 injections to assess instrument stability.

Chromatographic separation was performed on an Agilent SB-C18 column (2.1 mm × 100 mm, 1.8 μm) maintained at 40 °C. The mobile phase consisted of water (A) and acetonitrile (B), both with 0.1% formic acid, at a flow rate of 0.35 mL/min, using a gradient elution program as follows: 0–1 min, 5% B; 1–9 min, 5–95% B linear; 9–11 min, 95% B; 11–11.1 min, 95–5% B; 11.1–14 min, 5% B for re-equilibration. The injection sequence was fully randomized across all samples to minimize systematic bias. Mass spectrometry detection was conducted using a QTRAP® 6500+ system with an electrospray ionization source operating in both positive and negative modes (ESI+/−). Ion spray voltages were set to 5500 V (positive) and − 4500 V (negative), with a source temperature of 500 °C. The instrument was operated at unit resolution (0.7 Da FWHM) for both Q1 and Q3 in multiple reaction monitoring (MRM) mode, with a dwell time of 20 ms per transition. Data acquisition utilized Multiple Reaction Monitoring (MRM) mode, with metabolite-specific transitions and optimized collision energies established from the Metware Database (MWDB). A scheduled MRM algorithm was applied to monitor each transition within a defined retention window (Chen et al., 2013).

Metabolites were identified by matching their MS/MS spectra and retention times against the MWDB. All identified polyphenols, along with their molecular formulas, MRM transitions, CAS numbers, and identification confidence levels, are provided in Supplementary Table S1. According to the Metabolomics Standards Initiative (MSI) and COSMOS guidelines, compound annotations were assigned to Level 1, Level 2, or Level 3: Level 1 (MS/MS and retention time match score ≥ 0.7), Level 2 (match score 0.5–0.7), and Level 3 (Q1, Q3, retention time, declustering potential, and collision energy matched). The specific level for each metabolite is listed in Supplementary Table S1. Relative quantification was based on normalized peak areas, adjusted for the internal standard and sample weight; no semi-quantitative values (e.g., summed concentrations per phenolic class) were calculated.

### 2.5 RNA sequencing and transcriptomic data analysis

Total RNA was isolated from kiwifruit flesh samples (three biological replicates per variety) using TRIzol reagent (Invitrogen, Beijing, China). RNA integrity and concentration were assessed on an Agilent 2100 Bioanalyzer (Agilent Technologies, Santa Clara, CA, USA). Library construction and RNA sequencing were performed by Berry Hekang Biotech (Beijing, China). Sequencing libraries were prepared with the NEBNext Ultra™ RNA Library Prep Kit (NEB, Ipswich, MA, USA) following the manufacturer's protocol. Paired-end sequencing (2 × 150 bp) was carried out on an Illumina HiSeq 2500 platform (Illumina, Shanghai, China). Raw reads were filtered to remove adaptor sequences and low-quality reads using Cutadapt (v1.18) and FastQC (v0.11.8) for quality control.

Clean reads were aligned to the kiwifruit reference genome of *Actinidia chinensis* (Red5 v3; accessible at https://kiwifruitgenome.org/) using HISAT2 (v2.1.0). Gene expression levels were quantified as fragments per kilobase of transcript per million mapped reads (FPKM) using StringTie (v2.1.3). Differential expression analysis between variety pairs was performed using Cuffdiff (v2.2.1) with thresholds of |log₂(fold change)| ≥ 1 and *p* < 0.05.

Functional annotation of genes was conducted against multiple databases, including Kyoto Encyclopedia of Genes and Genomes (KEGG), Gene Ontology (GO), Pfam, Swiss-Prot, NCBI non-redundant protein sequences (Nr), and Clusters of Orthologous Groups (KOG/COG). GO enrichment analysis was carried out using the GOseq R package (v2.18.0), which applies the Wallenius non-central hypergeometric distribution. KEGG pathway enrichment was analyzed with KOBAS 2.0. For transcription factor (TF) analysis, the annotated gene set was screened against the Plant Transcription Factor Database (PlantTFDB v5.0) to identify MYB family members. The raw RNA-seq data have been deposited in the China National GeneBank (CNGB) under project accession No. CNP0008694.

### 2.6 Unsupervised and supervised statistical analysis

#### 2.6.1 Unsupervised multivariate analysis

Statistical analyses were conducted to explore metabolic variation among the five kiwifruit varieties. Following unit variance scaling of the normalized metabolite data, unsupervised Principal Component Analysis (PCA) was performed using the stats package (v3.5.1) in R (v3.5.1), to visualize overall sample clustering. The abundance patterns of all metabolites across samples were also examined using Hierarchical Cluster Analysis (HCA), presented as a circular heatmap with the circlize package (v0.4.15) in R (v4.2.0) (Chanana et al., 2020; Chong & Xia, 2018).

Differential metabolites between each pair of varieties were identified by applying a dual-threshold criterion of |log₂FC| ≥ 1.0 and a *p*-value <0.05 from Student's *t*-tests, with False Discovery Rate (FDR) correction (Colquhoun, 2014). Radar charts visualizing the top 20 significantly altered phenolic compounds for pairwise variety comparisons were generated using the fmsb package (version 0.7.3) in R (version 4.2.0). To functionally interpret these results, a metabolite classification bubble chart and a classification scatter plot were generated using differential metabolite data, incorporating classification levels, log₂FC values, and significance metrics (ggplot2 v3.4.0, R v4.2.2). For key differential metabolites, a bar chart of the top 20 compounds by |log₂FC| was created for visualization (ggplot2 v3.3.0, R v3.5.1). Pairwise correlations between differential metabolites were analyzed and visualized using a combined heatmap and scatter plot (ComplexHeatmap v2.12.0, R v4.2.0) (Chong & Xia, 2018).

For comparisons of targeted biochemical traits (e.g., phenolic compounds, radical-scavenging capacity), significant differences among varieties were determined using Duncan's multiple range test, with significance set at *p* < 0.05. Venn diagrams were created using the online EVenn platform to illustrate shared and unique metabolites across comparison groups (https://www.bic.ac.cn/EVenn/#/) (Yang et al., 2024).

#### 2.6.2 Supervised orthogonal partial least squares-discriminant analysis (OPLS-DA)

Orthogonal Partial Least Squares-Discriminant Analysis (OPLS-DA) was employed as a supervised multivariate method to maximize separation between the five kiwifruit varieties and to identify metabolites responsible for group discrimination (Thévenot et al., 2015). The model separates predictive variation in the metabolite data (X-matrix) that is correlated with class labels (Y-matrix) from orthogonal (non-predictive) variation, thereby improving model interpretability.

Log₂-transformed and mean-centered relative quantification data were used as the input. The analysis was performed using the MetaboAnalystR package (version 1.0.1) within the R statistical environment (version 3.5.1). Default parameters were applied for model construction. Model quality was assessed using the explained variance parameters R^2^X (X-matrix) and R^2^Y (Y-matrix), and the predictive parameter Q^2^. A model with Q^2^ > 0.5 was considered robust. A permutation test (*n* = 200) was conducted for validation by randomly shuffling the class labels to create null models; a significant model (*p* < 0.05) was indicated when the original Q^2^ value was higher than those from all permuted models (Eriksson et al., 2013). Metabolites with a Variable Importance in Projection (VIP) score > 1.0 were considered significant contributors to group separation.

## 3 Results

### 3.1 Phenolic metabolite diversity and varietal accumulation patterns

**Fig. 1:**
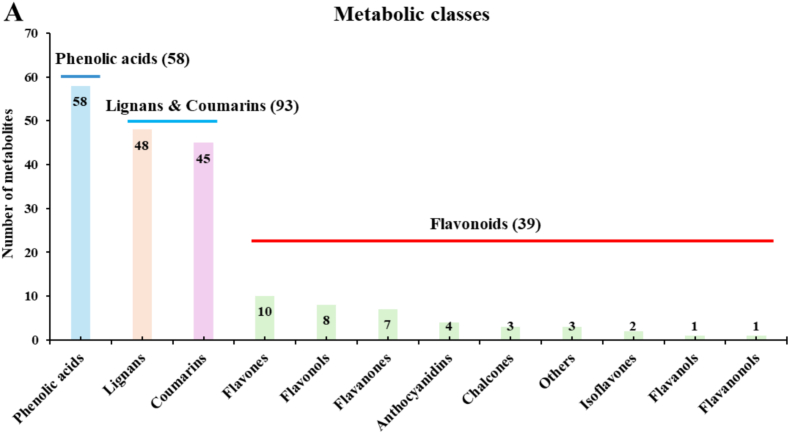

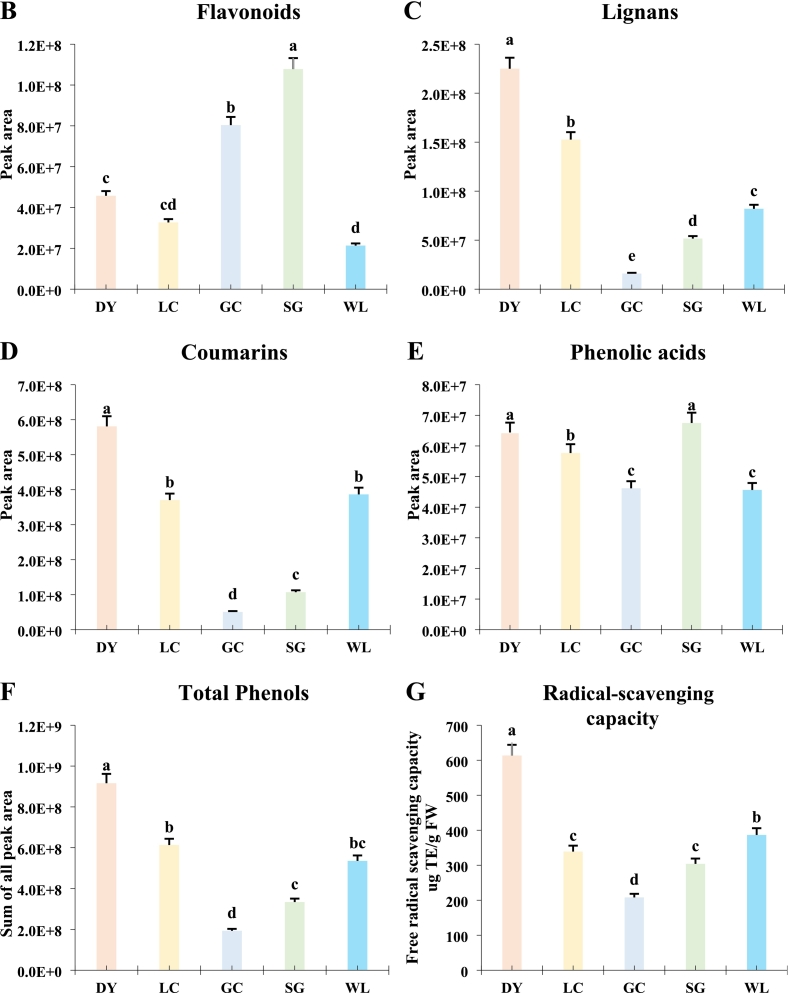
Phenolic metabolite composition across five kiwifruit varieties: LC (*A. arguta* ‘Maolvfeng’), WL (wild *A. chinensis*), GC (*A. chinensis* ‘Guichang’), SG (*A. chinensis* ‘SunGold’), and DY (*A. arguta* ‘Danyang flat’). (A) Number of metabolites identified within each major phenolic class. (B–E) Total content of (B) flavonoids, (C) lignans, (D) coumarins, (E) phenolic acids, (F) total phenols (y-axis denotes sum of peak areas of compounds), and (G) DPPH● free radical scavenging capacity μg TE/g FW. Values are mean ± SE (*n* = 3). Different lowercase letters indicate significant differences among varieties (Duncan's test, *p* < 0.05).

Widely targeted metabolomic analysis identified 190 phenolic metabolites across the five kiwifruit varieties (Fig. 1A). Phenolic acids were the most abundant class (58 compounds), followed by lignans (48) and coumarins (45). Flavonoids were represented by several sub-classes, primarily flavones (10), flavonols (8), and flavanones (7).

Comparative analysis of the relative abundance of phenolic classes revealed distinct accumulation patterns among the varieties (Fig. 1B–E). The total flavonoid content was highest in ‘SunGold’ (SG) (Fig. 1B). In contrast, the *A. arguta* variety ‘Danyang flat’ (DY) accumulated significantly higher levels of both lignans (Fig. 1C) and coumarins (Fig. 1D) compared to the *A. chinensis* varieties. Among *A. chinensis* varieties, the WL showed higher accumulation of lignans and coumarins than GC and SG. The phenolic acids were significantly higher in SG and DY than in the other varieties (Fig. 1E). DY also exhibited the highest total phenols (Fig. 1F) and the strongest DPPH● radical scavenging capacity (613.4 μg TE/g FW), followed by WL, LC, SG, and GC (Fig. 1G), indicating a clear link between phenolic abundance and radical-scavenging capacity. These results establish distinct phenolic profiles: SG is rich in flavonoids, whereas DY is a notable accumulator of lignans and coumarins, and overall radical-scavenging capacity.

### 3.2 Metabolic fingerprints clearly distinguish species and varieties

**Fig. 2:**
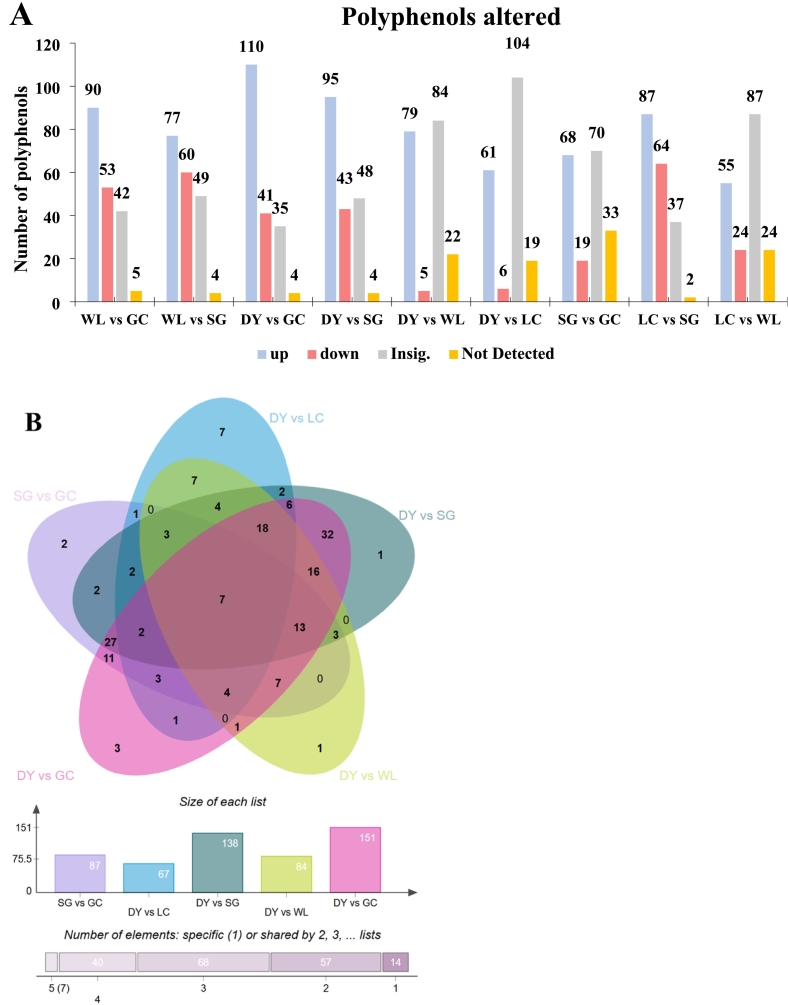

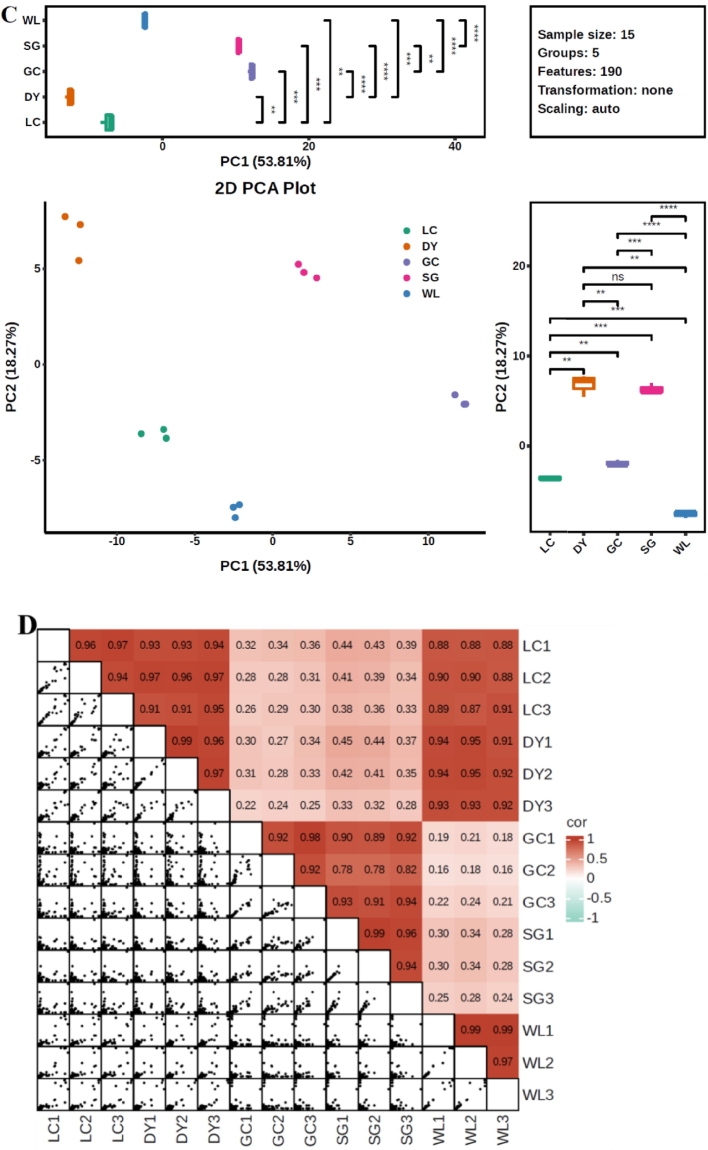
Multivariate analysis reveals species-level metabolic separation among five kiwifruit varieties: LC (*A. arguta* ‘Maolvfeng’), WL (wild *A. chinensis*), GC (*A. chinensis* ‘Guichang’), SG (*A. chinensis* ‘SunGold’), and DY (*A. arguta* ‘Danyang flat’). (A) Differential metabolite counts (up-regulated, down-regulated, insignificant, not detected) for nine pairwise comparisons. (B) Venn diagram of shared and unique differential metabolites across five comparison groups. (C) PCA scores plot showing varietal clustering with boxplots of PC1 and PC2 scores; asterisks indicate significant inter-group differences. (D) Correlation matrix with a heatmap of Pearson coefficients (upper right) and scatter plots of selected metabolite pairs (lower left).

**Fig. 3:**
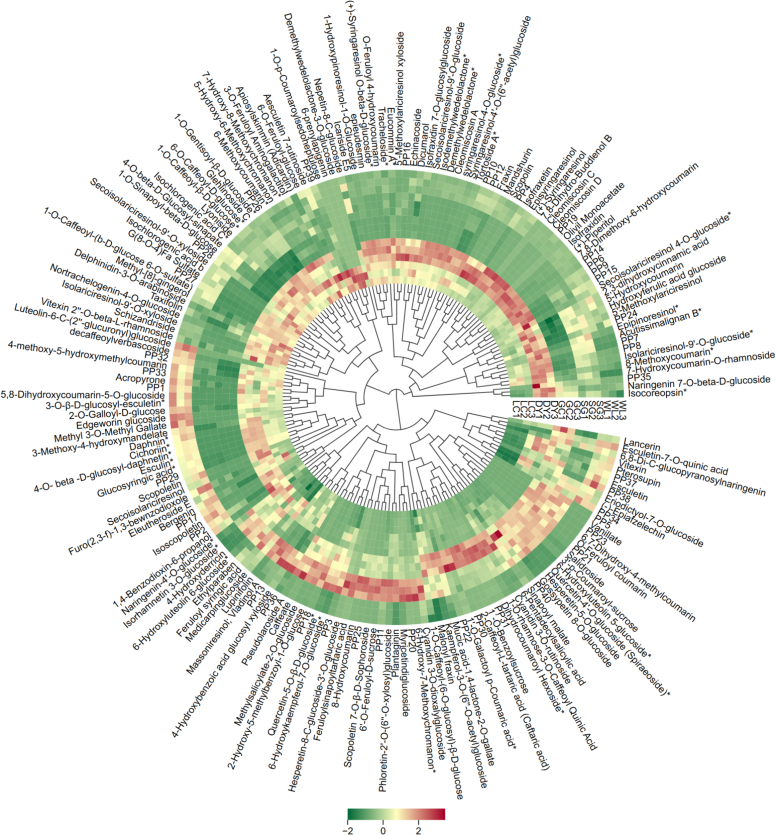
Hierarchical cluster analysis (HCA) heatmap of 190 phenolic metabolites across five kiwifruit varieties: LC (*A. arguta* ‘Maolvfeng’), WL (wild *A. chinensis*), GC (*A. chinensis* ‘Guichang’), SG (*A. chinensis* ‘SunGold’), and DY (*A. arguta* ‘Danyang flat’). Rows represent *Z*-score-normalized metabolites and columns represent biological replicates. PP1-PP40 compounds names are represented in supplementary Table S3. The colour gradient (green to red) indicates relative abundance from low to high. Clustering reveals distinct metabolite accumulation patterns among varieties. (For interpretation of the references to colour in this figure legend, the reader is referred to the web version of this article.)

Multivariate analysis revealed significant metabolic divergence among the five kiwifruit varieties, driven primarily by species. Pairwise comparisons between *A. arguta* (DY) and *A. chinensis* varieties showed the highest number of differential metabolites, with DY vs. GC having 151 compounds (Fig. 2A). In contrast, comparisons within the same species (e.g., DY vs. LC) yielded only 67 differential metabolites, indicating closer metabolic relationships. A Venn diagram identified seven phenolic metabolites consistently present across all comparisons (Fig. 2B). PCA captured 53.8% (PC1) and 18.3% (PC2) of total variance, with clear separation of *A. arguta* (DY, LC) from *A. chinensis* varieties (GC, SG, WL) along PC1 (Fig. 2C). Correlation analysis showed a strong positive correlation among biological replicates confirmed to be high reproducibility (Fig. 2D). HCA further illustrated distinct accumulation patterns: a block of metabolites highly abundant in DY corresponded to lignans and coumarins, while a separate block enriched in SG aligned with flavonoids (Fig. 3). Together, these results demonstrate that species is the primary determinant of metabolic divergence, with high reproducibility across replicates, establishing a robust foundation for identifying variety-specific phenolic fingerprints.

### 3.3 Supervised OPLS-DA validates separation and identifies key discriminatory metabolites

**Fig. 4:**
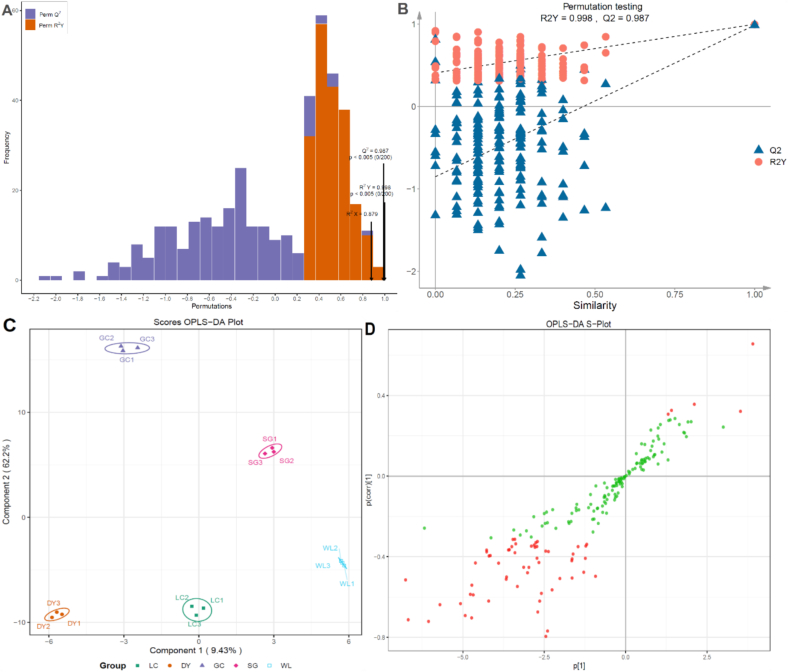
Orthogonal Partial Least Squares-Discriminant Analysis (OPLS-DA) validates varietal separation and identifies key discriminatory metabolites. Phenolic profiles across five kiwifruit varieties were analyzed: LC (*A. arguta* ‘Maolvfeng’), WL (wild *A. chinensis*), GC (*A. chinensis* ‘Guichang’), SG (*A. chinensis* ‘SunGold’), and DY (*A. arguta* ‘Danyang flat’). (A) Model validation metrics (R^2^X, R^2^Y, Q^2^) showing effective predictive performance (Q^2^ > 0.5). (B) Permutation test (*n* = 200) confirming model robustness, with all permuted Q^2^ values (red) lower than the original model. (C) Scores plot showing clear separation between varieties along the predictive component (t[1]); ellipses represent 95% confidence intervals. (D) S-plot identifying influential metabolites; compounds with VIP > 1.0 are highlighted in red. The model confirms robust varietal separation and pinpoints specific reliable chemomarkers. (For interpretation of the references to colour in this figure legend, the reader is referred to the web version of this article.)

A supervised OPLS-DA model was constructed and validated to rigorously discriminate the phenolic profiles among the five kiwifruit varieties. The model demonstrated excellent fit and predictive reliability, with high explained variance (R^2^X, R^2^Y, *P* < 0.005) and a predictive parameter Q^2^ > 0.5 (Fig. 4A). Its robustness was confirmed by a 200-permutation test, which showed no overfitting, as all permuted Q^2^ values fell significantly below the original model's value (Fig. 4B). The OPLS-DA scores plot revealed clear varietal separation, predominantly along the first predictive component 2 (t[1], 9.3% variance) captured intra-group variation, while the second orthogonal component 2 (to[1], 62.2% variance), which effectively partitioned the two *A. arguta* varieties (DY, LC) from the three *A. chinensis* varieties (GC, SG, WL) (Fig. 4C). This supervised separation was consistent with prior unsupervised analyses. The corresponding S-plot pinpointed the metabolites most responsible for this discrimination, with compounds having a Variable Importance in Projection (VIP) > 1.0 highlighted in red (Fig. 4D). These key discriminatory markers, encompassing specific phenolic acids, lignans, and flavonoids, definitively characterize the unique metabolic fingerprint of each variety. Thus, the OPLS-DA model not only validates the robust metabolic separation between species and varieties but also identifies a reliable set of chemomarkers that can serve as targets for quality assessment and breeding selection.

### 3.4 Radar chart analysis reveals enrichment of lignans and coumarins in *A. arguta*

**Fig. 5:**
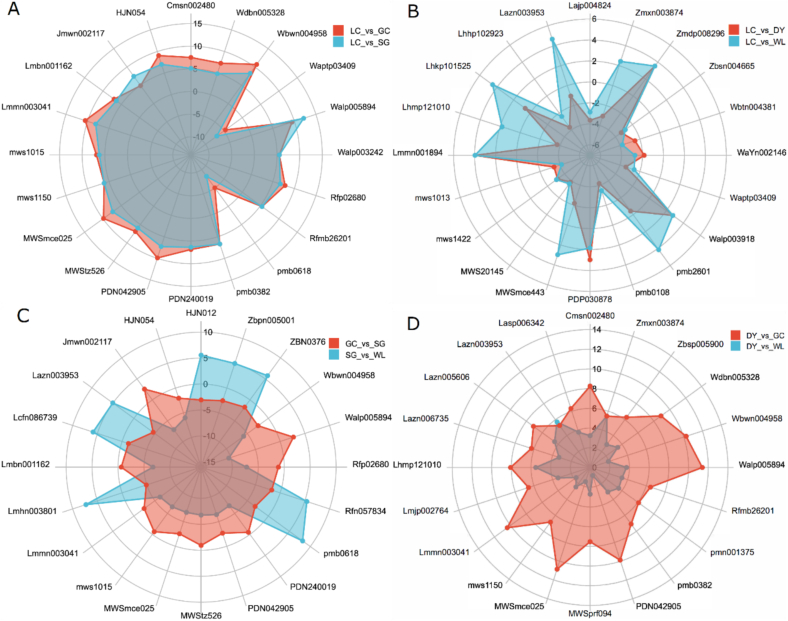
Radar chart analysis highlights extreme inter-species differences and enrichment of lignans/coumarins in *A. arguta*. Profiles of the top 20 significantly altered phenolic compounds (Log₂FC) across pairwise comparisons of five kiwifruit varieties: LC (*A. arguta* ‘Maolvfeng’), WL (wild *A. chinensis*), GC (*A. chinensis* ‘Guichang’), SG (*A. chinensis* ‘SunGold’), and DY (*A. arguta* ‘Danyang flat’). Each axis represents one compound, with the distance from the center indicating its relative value. (A) LC vs. GC (red) and LC vs. SG (blue). (B) LC vs. DY (red) and LC vs. WL (blue). (C) GC vs. SG (red) and SG vs. WL (blue). (D) DY vs. GC (red) and DY vs. WL (blue). The radar shapes reveal that inter-species differences are more pronounced than intra-species variation. (For interpretation of the references to colour in this figure legend, the reader is referred to the web version of this article.)

Radar chart analysis of the top 20 differential metabolites revealed distinct regulatory patterns among varieties (Fig. 5). Inter-species comparisons showed the most pronounced effects: radar profiles for LC (*A. arguta*) vs. GC and SG were nearly superimposable, with strong up-regulation of lignans and coumarins (e.g., schizandriside Wbwn004958, fraxin MWSmce025; log₂FC up to 10.7) (Fig. 5A, Table 1). In contrast, intra-species comparisons (LC vs. DY, both *A. arguta*) displayed a symmetrical pattern with reciprocal up/down-regulation (Fig. 5B, red), while comparisons among *A. chinensis* varieties showed moderate, variety-specific shapes (Fig. 5C).

**Table 1:** Top 20 significantly differential phenolic metabolites between kiwifruit varieties: Comparisons of LC (*A. arguta* ‘Maolvfeng’) vs GC (*A. chinensis* ‘Guichang’) and LC vs SG (*A. chinensis* ‘SunGold’).

Serial No	Index	Compounds	Class	LC vs GC			LC vs SG		
				Fold Change	Log2FC	Type	Fold Change	Log2FC	Type
1	Wbwn004958	Schizandriside	Lignans	1660.8	10.7	up	306.8	8.3	up
2	Lmmn003041	Isolariciresinol-9’-O-xyloside	Lignans	1497.9	10.5	up	277.4	8.1	up
3	PDN042905	G(8-O-4)Fa Sulfate	Phenolic acids	964.9	9.9	up	155.5	7.3	up
4	MWSmce025	Fraxin	Coumarins	932.5	9.9	up	164.0	7.4	up
5	Walp005894	Cleomiscosin C	Coumarins	710.3	9.5	up	4599.1	12.2	up
6	HJN054	Secoisolariciresinol-9’-O-xyloside	Lignans	538.4	9.1	up	130.7	7.0	up
7	Rfp02680	3-O-β-D-glucosyl-esculetin⁎	Coumarins	231.6	7.9	up	107.5	6.7	up
8	Cmsn002480	Secoisolariciresinol-9’-O-glucoside	Lignans	183.8	7.5	up	34.8	5.1	up
9	Wdbn005328	3,3’-Bis(3,4-dihydro-4-hydroxy-6,8-dimethoxy-2H-1-benzopyran)	Lignans	157.1	7.3	up	29.1	4.9	up
10	Lmbn001162	Cichoriin⁎	Coumarins	124.9	7.0	up	81.3	6.3	up
11	PDN240019	4-O- beta -D-glucosyl-daphnetin⁎	Coumarins	118.2	6.9	up	83.6	6.4	up
12	MWStz526	Daphnin⁎	Coumarins	117.9	6.9	up	61.8	5.9	up
13	mws1015	Esculin⁎	Coumarins	116.4	6.9	up	75.1	6.2	up
14	pmb0382	O-Feruloyl 4-hydroxycoumarin	Coumarins	102.3	6.7	up	102.3	6.7	up
15	mws1150	Echinacoside	Phenolic acids	71.1	6.2	up	71.1	6.2	up
16	Jmwn002117	2,6-dimethoxybenzene-1,4-diol 1-O-β-D-glucopyranoside	Phenolic acids	30.0	4.9	up	177.4	7.5	up
17	Walp003242	Isofraxidin 7-O-glucosylglucoside	Coumarins	46.2	5.5	up	46.2	5.5	up
18	Rfmb26201	Syringaresinol-4’-O-(6″-acetyl)glucoside	Lignans	42.5	5.4	up	42.5	5.4	up
19	Waptp03409	6-Hydroxy-7-Methoxychromanon⁎	Coumarins	0.041	−4.6	down	0.008	−6.9	down
20	pmb0618	Hesperetin-8-C-glucoside-3’-O-glucoside	Flavanones	0.030	−5.0	down	0.003	−8.2	down
**Serial No**	**Index**	**Compounds**	**Class**	**LC vs DY**			**LC vs WL**		
				**Fold Change**	**Log2FC**	**Type**	**Fold Change**	**Log2FC**	**Type**
1	Lazn003953	Erythro-Guaiacylglycerol-β-dihydroconiferyl Ether glucoside	Lignans	0.48	−1.05	down	25.29	4.66	up
2	Lhkp101525	Apiosylskimmin (Adicardin)	Coumarins	1.56	0.65	insig	22.33	4.48	up
3	pmb2601	7-Hydroxycoumarin-O-rhamnoside	Coumarins	0.75	−0.42	insig	17.67	4.14	up
4	Lmmn001894	Demethylwedelolactone-3-O-glucoside	Coumarins	15.65	3.97	up	15.65	3.97	up
5	Zmdp008296	6-prenylapigenin	Flavones	11.57	3.53	up	11.57	3.53	up
6	MWSmce443	4-Hydroxyderricin	Chalcones	0.22	−2.18	down	7.91	2.98	up
7	Walp003918	1-O-p-Coumaroylsedoheptulose	Phenolic acids	6.79	2.76	up	6.79	2.76	up
8	Zmxn003874	7,8-Dihydro-Buddlenol B	Lignans	0.12	−3.06	down	5.39	2.43	up
9	PDP030878	2-(3,4-dihydroxyphenyl)-6,7-dihydroxy-4H-chromen-4-one	Flavones	7.87	2.98	up	3.61	1.85	up
10	Lhmp121010	Olivil Monoacetate	Lignans	0.08	−3.71	down	3.52	1.81	up
11	Lhhp102923	(7′*R*,8′*R*)-7′8’-dihydro-7′-(5′-hydroxy-3′-methoxphenyl)-3-methoxy-8′-methyl-1-(*E*)-propenylbenzof-uran	Lignans	0.08	−3.69	down	0.19	−2.41	down
12	Waptp03409	6-Hydroxy-7-Methoxychromanon⁎	Coumarins	0.09	−3.40	down	0.17	−2.57	down
13	WaYn002146	2-(Hydroxymethyl)Phenyl 2-O-Beta-d-Glucopyranosyl-Beta-D-Glucopyranoside	Phenolic acids	0.28	−1.83	down	0.15	−2.71	down
14	Zbsn004665	Vitexin	Flavones	0.10	−3.32	down	0.14	−2.85	down
15	Lajp004824	Medicarpinglucoside	Isoflavone	0.08	−3.65	down	0.14	−2.86	down
16	mws1422	(−)-Epiafzelechin	Flavanols	0.11	−3.18	down	0.12	−3.04	down
17	pmb0108	Feruloyl syringic acid	Phenolic acids	0.06	−4.17	down	0.09	−3.45	down
18	MWS20145	Eriodictyol-7-O-glucoside	Flavanones	0.07	−3.84	down	0.08	−3.64	down
19	Wbtn004381	1,3,6,8-tetrahydroxy-2,5-dimethoxyxanthen-9-one	Flavonoids	0.18	−2.49	down	0.07	−3.75	down
20	mws1013	Esculetin	Coumarins	0.10	−3.39	down	0.06	−4.15	down

⁎ means isomers.

The most asymmetric profiles involved DY: DY vs. GC exhibited extreme outward projections for Walp005894 (cleomiscosin C, log₂FC 11.4), MWSmce025 (fraxin, 10.9), Lmmn003041 (isolariciresinol-9′-O-xyloside, 10.4), Wbwn004958 (schizandriside, 10.3), and PDN042905 (G(8-O-4)Fa sulfate, 9.9) (Fig. 5D, red). DY vs. WL mirrored this pattern at lower magnitude (Fig. 5D, blue). Collectively, radar analysis confirms that species-level divergence dictates the major metabolic framework, with *A. arguta* varieties (especially DY) showing extreme up-regulation of lignans and coumarins, while varietal selection fine-tunes profiles in a more moderate manner.

### 3.5 Phenolic profile of ‘Danyang flat’ (DY) is distinctly enriched in lignans and coumarins relative to ‘SunGold’ (SG)

**Fig. 6:**
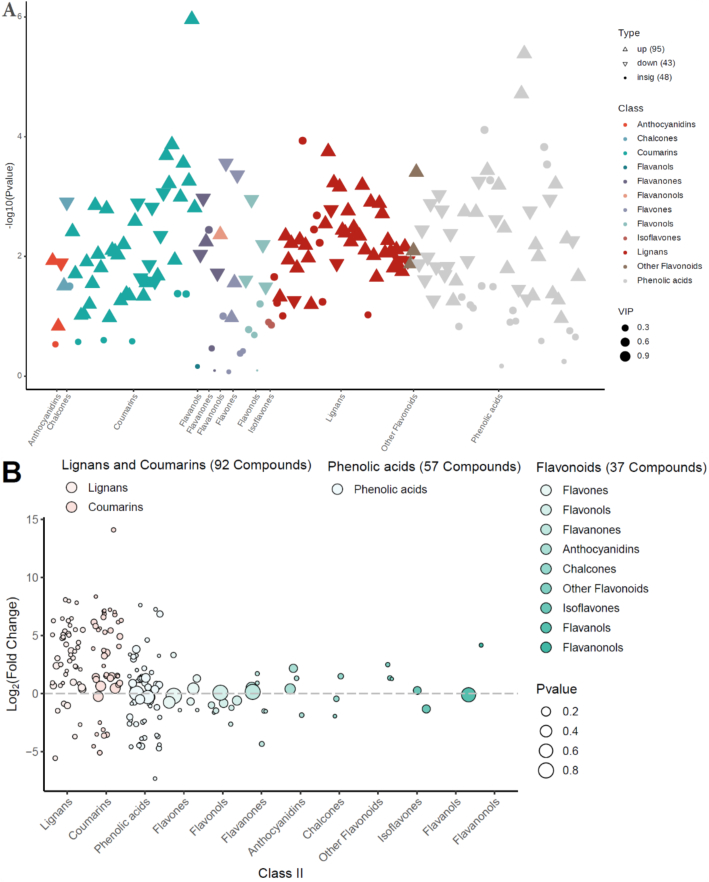

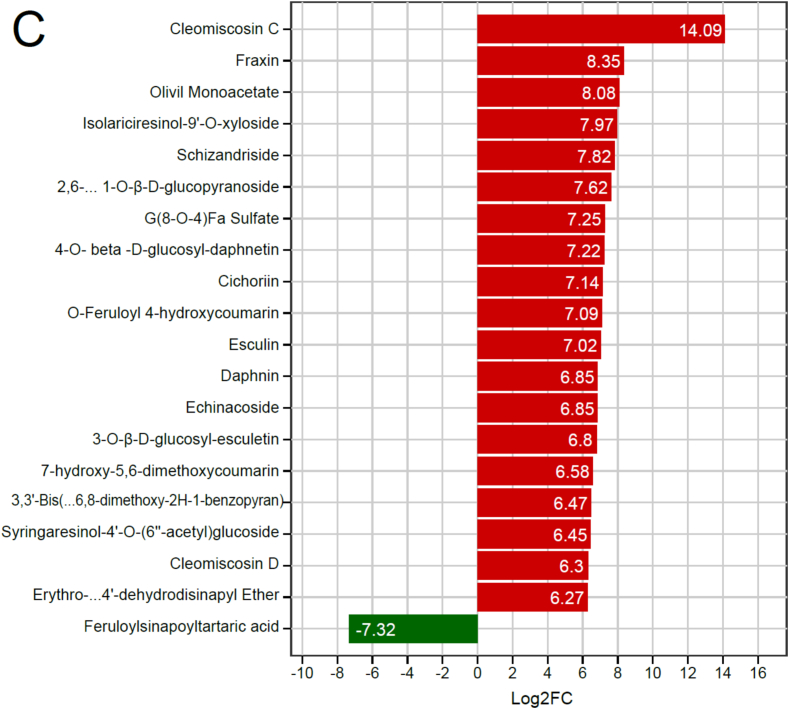
Differential phenolic profiles between ‘Danyang flat’ (DY, *Actinidia arguta*) and ‘SunGold’ (SG, *Actinidia chinensis*) kiwifruits. (A) Class-based significance of altered metabolites. Metabolite categories are plotted against -log10(*p*-value); triangles indicate up/down-regulation (VIP scaled to size), dots show non-significant changes. (B) Bubble chart of class-specific regulation, with metabolites arranged by subclass (x-axis) and log2(FC) (y-axis); bubble size reflects significance, and colors represent primary classes. (C) Top 20 metabolites ranked by absolute log2(FC); abbreviated names (indicated by “…”) are fully listed in Table 2. Bars show fold-change direction and magnitude in DY vs. SG (red = up, green = down). (For interpretation of the references to colour in this figure legend, the reader is referred to the web version of this article.)

To decipher the unique metabolic signature of the high-phenolic *A. arguta* variety DY, a targeted comparison was made with the commercial *A. chinensis* variety SG. Analysis of class significance revealed lignans and coumarins as the most dramatically altered phenolic groups between the two varieties (Fig. 6A). These classes showed the strongest statistical significance (−log₁₀(*p*-value)) and highest VIP scores, marked by prominent upward-pointing triangles indicating substantial up-regulation in DY. This class-specific enrichment was further detailed in the bubble chart (Fig. 6B), which illustrated that the most pronounced up-regulation occurred within specific subclasses, particularly furofuran lignans and simple coumarin glucosides. The concentration of large bubbles high on the log₂FC axis visually confirms DY's specialized accumulation of these compounds.

The scale of this divergence is quantified by the top 20 differential metabolites (Fig. 6C and Table 2). Notably, 19 of these were markedly more abundant in DY. The list was dominated by lignans and coumarins with high VIP > 1.17, confirming their role as key discriminators (Table 2). The coumarin cleomiscosin C (Walp005894) showed the most extraordinary up-regulation, with a fold change of 17,484 (log₂FC 14.09). Other major contributors included the coumarin fraxin (MWSmce025, log₂FC 8.35) and the lignans olivil monoacetate (Lhmp121010, log₂FC 8.08) and isolariciresinol-9’-O-xyloside (Lmmn003041, log₂FC 7.97). The single metabolite down-regulated in DY was the phenolic acid derivative feruloylsinapoyltartaric acid (Lmhn003801, log₂FC −7.32), which was more characteristic of the SG profile (Fig. 6C). This comparative analysis definitively establishes that the metabolic uniqueness of DY lies in its potent, coordinated up-regulation of specific lignan and coumarin biosynthetic pathways, creating a phenolic signature that is fundamentally distinct from the commercially dominant SG.

**Table 2:** Top 20 differential phenolic metabolites between DY (*A. arguta* ‘Danyang flat’) and SG (*A. chinensis* ‘SunGold’) kiwifruit varieties.

Serial No	Index	Compounds	Class II	VIP	*P*-value	Fold Change	Log2FC	Type
1	Walp005894	Cleomiscosin C	Coumarins	1.171	0.008	17,484.39	14.09	up
2	MWSmce025	Fraxin	Coumarins	1.171	0.000	326.88	8.35	up
3	Lhmp121010	Olivil Monoacetate	Lignans	1.171	0.006	269.93	8.08	up
4	Lmmn003041	Isolariciresinol-9’-O-xyloside	Lignans	1.171	0.002	250.59	7.97	up
5	Wbwn004958	Schizandriside	Lignans	1.171	0.001	226.17	7.82	up
6	Jmwn002117	2,6-dimethoxybenzene-1,4-diol 1-O-β-D-glucopyranoside	Phenolic acids	1.171	0.001	196.08	7.62	up
7	PDN042905	G(8-O-4)Fa Sulfate	Phenolic acids	1.170	0.002	152.31	7.25	up
8	PDN240019	4-O- beta -D-glucosyl-daphnetin⁎	Coumarins	1.171	0.001	148.73	7.22	up
9	Lmbn001162	Cichoriin⁎	Coumarins	1.171	0.000	141.38	7.14	up
10	pmb0382	O-Feruloyl 4-hydroxycoumarin	Coumarins	1.171	0.003	136.55	7.09	up
11	mws1015	Esculin⁎	Coumarins	1.170	0.000	130.07	7.02	up
12	MWStz526	Daphnin⁎	Coumarins	1.171	0.004	115.50	6.85	up
13	mws1150	Echinacoside	Phenolic acids	1.165	0.055	115.45	6.85	up
14	Rfp02680	3-O-β-D-glucosyl-esculetin⁎	Coumarins	1.170	0.009	111.26	6.80	up
15	Lmjp002764	Umckalin (7-hydroxy-5,6-dimethoxycoumarin)	Coumarins	1.168	0.021	95.61	6.58	up
16	Wdbn005328	3,3’-Bis(3,4-dihydro-4-hydroxy-6,8-dimethoxy-2H-1-benzopyran)	Lignans	1.171	0.001	88.61	6.47	up
17	Rfmb26201	Syringaresinol-4’-O-(6″-acetyl)glucoside	Lignans	1.171	0.001	87.62	6.45	up
18	Lasp006342	Cleomiscosin D	Coumarins	1.171	0.006	78.86	6.30	up
19	Lazn006735	Erythro-Guaiacylglycerol-β-O-4′-dehydrodisinapyl Ether	Lignans	1.171	0.007	77.14	6.27	up
20	Lmhn003801	Feruloylsinapoyltartaric acid	Phenolic acids	1.171	0.001	0.006	-7.32	down

⁎ means isomers.

### 3.6 Variety-specific accumulation patterns of key phenolic compounds

**Fig. 7:**
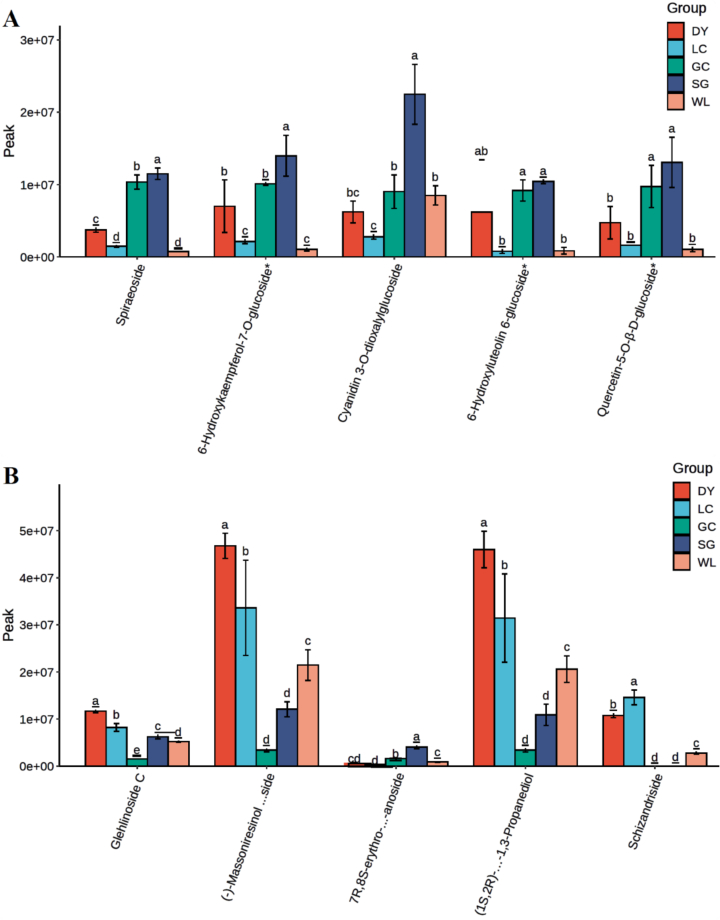

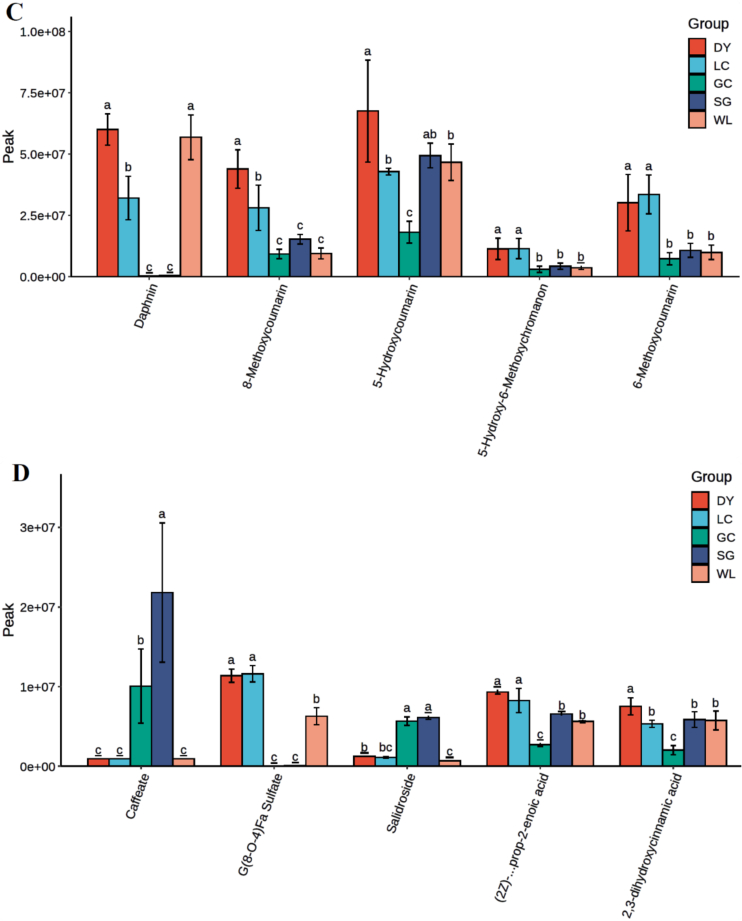
Abundance of key phenolic compounds across five kiwifruit varieties. Relative levels (peak area) of selected (A) flavonoids, (B) lignans, (C) coumarins, and (D) phenolic acids in LC (*A. arguta* ‘Maolvfeng’), WL (wild *A. chinensis*), GC (*A. chinensis* ‘Guichang’), SG (*A. chinensis* ‘SunGold’), and DY (*A. arguta* ‘Danyang flat’). Bars represent mean ± SE (*n* = 3). Different lowercase letters above bars indicate significant differences among varieties according to Duncan's multiple range test (*p* < 0.05).

Analysis of individual phenolic compounds confirmed distinct metabolic identities across varieties (Fig. 7). SG exhibited the highest flavonoid levels (e.g., cyanidin 3-O-dioxalylglucoside, 6-hydroxykaempferol-7-O-glucoside), while LC showed the lowest (Fig. 7A). In contrast, *A. arguta* varieties, especially DY, dominated lignan and coumarin accumulation, with far higher levels of compounds such as (−)-Massoniresinol 4’-O-β-D-glucopyranoside, 5-hydroxycoumarin, and 8-methoxycoumarin compared to all *A. chinensis* varieties (Fig. 7B, C). Phenolic acid profiles varied: SG and GC were richer in caffeate and salidroside, whereas LC and DY accumulated more G(8-O-4)Fa sulfate and 2,3-dihydroxycinnamic acid (Fig. 7D). These results confirm a fundamental divergence: *A. arguta* (especially DY) specializes in lignan/coumarin production, while *A. chinensis* varieties exhibit distinct flavonoid and phenolic acid profiles.

The integrated biosynthetic pathway (Supplementary Fig. S1) shows that shared precursors are differentially channeled: *A. arguta* directs flux toward lignans and coumarins (e.g., secoisolariciresinol, fraxin, esculin), whereas SG directs flux toward flavonoids (e.g., cyanidin 3-O-rutinoside). Varietal differences in intermediates like salidroside (SG-enriched) and 2,3-dihydroxycinnamic acid (DY-enriched) reflect variety-specific regulation of hydroxylation and glycosylation. These pathway-level insights confirm that genetic background determines the dominant phenolic biosynthetic route, providing a biochemical basis for varietal separation and metabolically informed breeding.

### 3.7 Transcriptomic analysis correlates MYB expression with phenolic variation

**Fig. 8:**
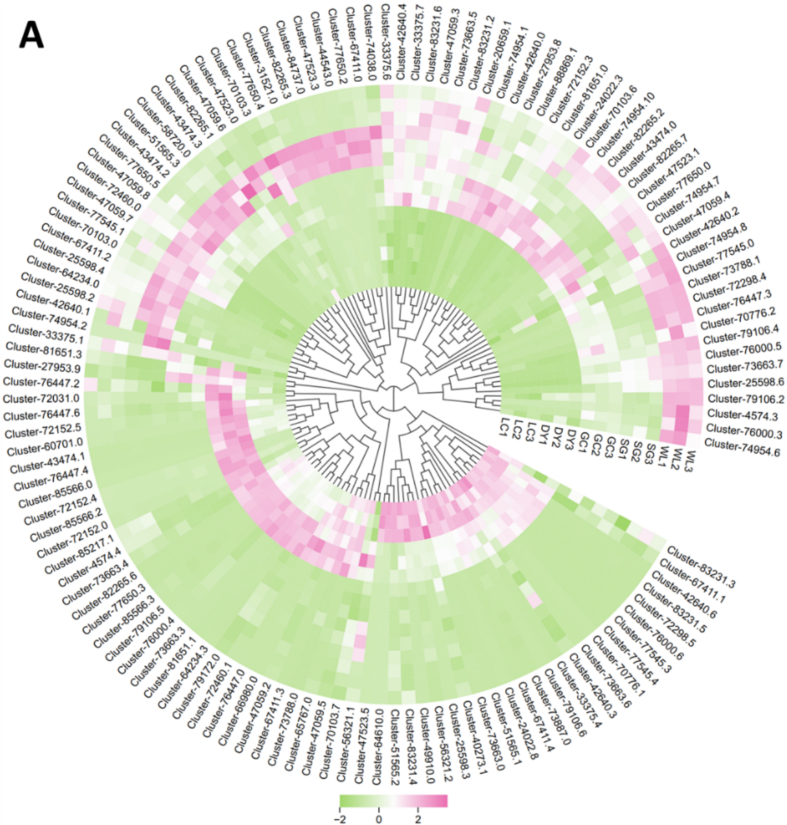

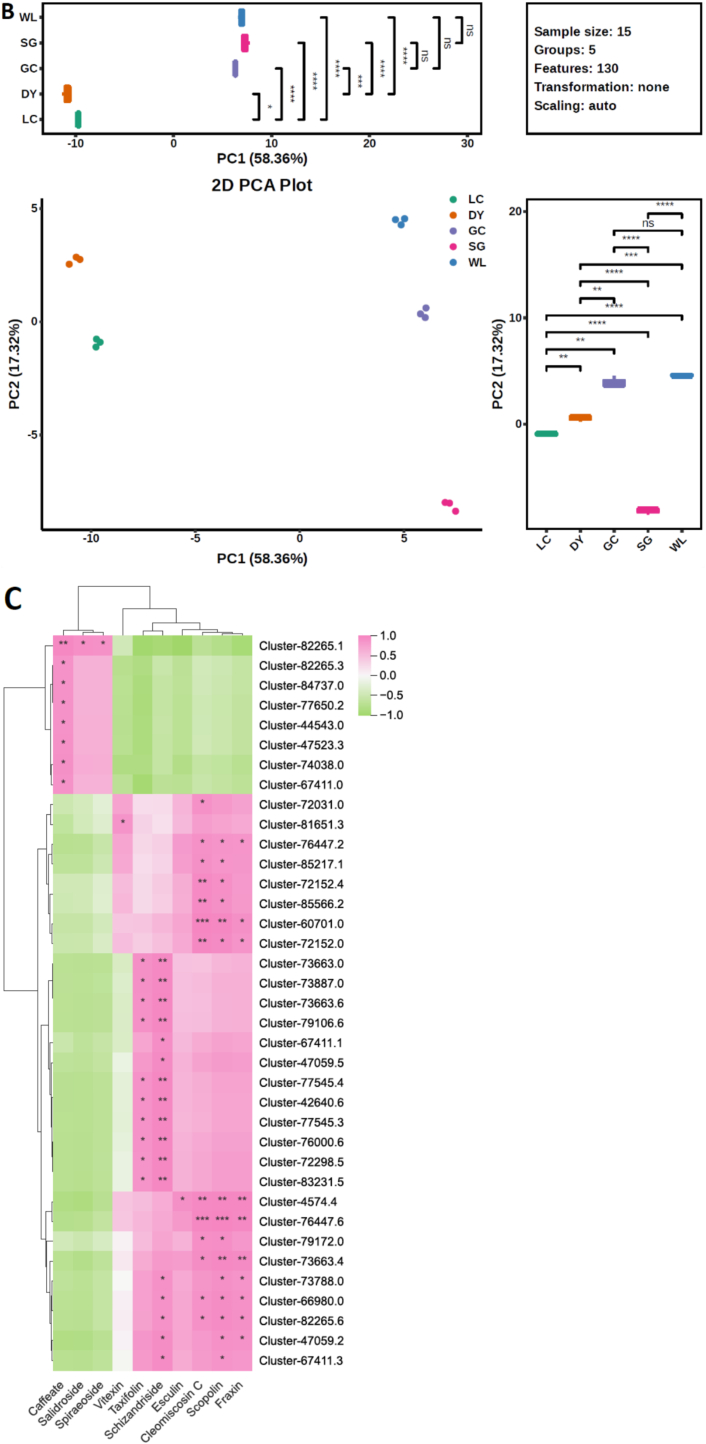
Integrative analysis of MYB transcription factors across kiwifruit varieties. (A) Hierarchical cluster analysis (HCA) of MYB gene expression profiles. (B) Principal component analysis (PCA) scores plot demonstrating varietal clustering based on MYB expression; boxplots of PC1 and PC2 scores are shown with asterisks indicating significant inter-group differences. (C) Correlation matrix heatmap displaying Pearson coefficients between MYB genes and phenolic metabolites (*p* < 0.05).

Transcriptomic analysis of MYB transcription factors reveals a species-specific regulatory architecture underlying the divergent phenolic profiles of kiwifruit. HCA of MYB gene expression (Fig. 8A) distinctly separated *A. arguta* (LC, DY) from *A. chinensis* (GC, SG, WL) varieties, identifying key regulatory candidates. Genes such as Cluster-76,447.6, Cluster-82,265.6, Cluster-4574.4, and Cluster-72,298.5 were uniquely upregulated in *A. arguta* (especially in DY), while Cluster-82,265.1 was highly abundant specifically in the flavonoid-rich ‘SunGold’ (SG). Principal component analysis (PCA, Fig. 8B) confirmed this strong transcriptional segregation, demonstrating that genetic background fundamentally shapes the MYB regulatory network. This differential expression directly correlates with the specialized phenolic output: *A. arguta*-enriched MYBs are implicated in lignan and coumarin biosynthesis, whereas the *A. chinensis*-specific cluster, particularly in SG, aligns with its flavonoid-dominant metabolism, providing a mechanistic link between transcriptional regulation and the production of distinct phytochemicals.

Pearson correlation analysis reveals significant association between MYB gene expression and phenolic metabolite levels, highlighting distinct, variety-specific regulatory networks (Fig. 8). In *A. arguta*, MYB genes such as Cluster-76,447.6, Cluster-82,265.6, Cluster-4574.4, and Cluster-72,298.5 strongly correlated with elevated lignans (e.g., schizandriside) and coumarins (e.g., fraxin, cleomiscosin C, scoplolin). In contrast, *A. chinensis*, particularly ‘SunGold’, exhibited MYB genes like Cluster-82,265.1 that were positively associated with phenolic acids and flavonoids, including caffeate and spiraeoside. These results indicate that species-specific phenolic profiles are correlated with MYB regulatory modules, which channel phenylpropanoid flux into distinct biosynthetic pathways, providing a transcriptional basis for the divergent accumulation of bioactive phenolics across kiwifruit varieties.

## 4 Discussion

The integrated metabolomic and transcriptomic analyses presented in this study reveal a clear species-level divergence in phenolic metabolism between *Actinidia arguta* and *A. chinensis*, underpinned by distinct MYB-mediated transcriptional regulation. The *A. arguta* variety ‘Danyang flat’ (DY) demonstrated a pronounced metabolic flux toward lignan and coumarin biosynthesis, whereas the *A. chinensis* variety ‘SunGold’ (SG) exhibited a flavonoid-dominant profile (Fig. 1, Fig. 3). This metabolic partitioning reflects both evolutionary adaptation and biochemical specialization within the *Actinidia* genus (Latocha et al., 2021; Rakha et al., 2025), offering new insights into the regulation and functional implications of phenolic diversity in kiwifruit.

The comparative metabolomic data establishes that DY channels phenylpropanoid intermediates preferentially into lignan and coumarin biosynthetic branches, as evidenced by the obvious fold changes of cleomiscosin C, fraxin, and isolariciresinol-9’-O-xyloside (Fig. 6C, Table 2). These metabolites exhibited log₂FC values exceeding 8–14. Such extreme up-regulation signifies a reprogramming of phenylpropanoid flux, likely reflecting species-specific enzymatic control at key branch points such as diligent proteins and *O*-methyltransferases (Ma & Constabel, 2019). In contrast, SG directs carbon flow toward flavonoid biosynthesis, producing elevated levels of cyanidin 3-O-rutinoside and 6-hydroxykaempferol-7-O-glucoside (Fig. 7A). This trade-off between lignan/coumarin and flavonoid accumulation exemplifies the metabolic plasticity of the phenylpropanoid pathway, where competition for shared precursors such as p-coumaroyl-CoA and ferulic acid determines the dominant phenolic class (Rahim et al., 2023; Vogt, 2010). Precision engineering of carbon partitioning has been proposed as a strategy to enhance crop resilience and redirect metabolic flux toward desired compounds (Zhao et al., 2025).

From an evolutionary perspective, the divergent phenolic strategies of *A. arguta* and *A. chinensis* may represent adaptive responses to distinct ecological pressures. Lignans and coumarins are often associated with defense against oxidative and microbial stress, UV protection, and allelopathic interactions (Ninkuu et al., 2025; Rao & Zheng, 2025). The elevated accumulation of these compounds in DY may thus confer enhanced resilience in its native temperate habitats. The superior radical-scavenging capacity of DY (613.4 μg TE/g FW; Fig. 1G) correlates with its lignan- and coumarin-rich profile, consistent with the high radical-scavenging potential of these compounds, which can donate hydrogen atoms and stabilize phenoxyl radicals through resonance (Kumar & Pandey, 2013; Rao, Wang, et al., 2025). Conversely, the flavonoid-dominant SG reflects a metabolic orientation toward pigmentation and photoprotection, traits often selected during domestication for fruit appearance and consumer preference (Duan et al., 2025).

The transcriptomic data provides an explanation for the observed metabolic divergence. Distinct clusters of MYB transcription factors were differentially expressed between *A. arguta* and *A. chinensis* (Fig. 8A). In DY, MYB genes such as Cluster-76,447.6, Cluster-82,265.6, and Cluster-72,298.5 were strongly upregulated and positively correlated with lignan and coumarin accumulation (Fig. 8C). These MYBs likely act as activators of downstream structural genes, which catalyze key steps in lignan biosynthesis. In contrast, SG exhibited high expression of Cluster-82,265.1, which correlated with flavonoid and phenolic acid accumulation, suggesting a role analogous to *AtMYB12* or *VvMYBF1*, known regulators of flavonol biosynthesis in *Arabidopsis* and grapevine (Czemmel et al., 2009; Luo et al., 2008; Pratyusha & Sarada, 2022; F. Wang et al., 2016). The strong correlation between MYB expression and metabolite abundance underscores the transcriptional control of phenylpropanoid flux partitioning, where MYB family members act as molecular switches directing carbon flow into specific branches of the pathway. This regulatory modularity mirrors patterns observed in other fruit crops, such as apple and blueberry, where distinct MYB subgroups orchestrate lignin, flavonoid, and coumarin biosynthesis (G. He et al., 2023; Thakur & Vasudev, 2022).

The discovery of a lignan- and coumarin-dominant phenotype in DY has significant implications for food chemistry and human nutrition. Lignans, such as isolariciresinol and syringaresinol derivatives, are well-established phytoestrogens with antioxidant, anti-inflammatory, and cardioprotective properties (Osmakov et al., 2022). They are also metabolized by gut microbiota into enterolignans, which exhibit prebiotic and hormone-modulating effects (Burgberger et al., 2025; Osmakov et al., 2022). Coumarins, including fraxin, daphnin, and cleomiscosin C, possess potent radical-scavenging and anti-inflammatory activities, and some have been reported to modulate glucose metabolism and vascular function (Kumari et al., 2023; Singh et al., 2025). The high abundance of these compounds in DY thus enhances its potential as a functional food with broad nutraceutical relevance. Compared to the flavonoid-centric antioxidant narrative commonly associated with kiwifruit, the lignan/coumarin enrichment in DY represents a distinct phytochemical niche, expanding the functional diversity of *Actinidia* species. Moreover, the synergistic antioxidant effects between lignans and coumarins may contribute to the exceptionally high DPPH● radical scavenging capacity observed in DY (Fig. 1G), as these compounds can regenerate each other's radical intermediates through redox cycling (Burgberger et al., 2025; Singh et al., 2025). Beyond nutrition, the identified key discriminatory metabolites (VIP > 1.0; Fig. 4D) and their associated MYB regulators provide a dual biomarker framework for targeted breeding and metabolic engineering. The strong species-level separation observed in OPLS-DA and PCA models confirms that phenolic composition is a reliable chemotaxonomic marker within *Actinidia*. These biomarkers can be exploited to develop cultivars with tailored phytochemical profiles — either lignan/coumarin-enriched for enhanced nutritional and functional quality, or flavonoid-rich for pigmentation and sensory appeal. Integrating metabolomic and transcriptomic markers into breeding pipelines aligns with current trends in precision horticulture, where genomic selection is guided by metabolite-based quality indices (Duan et al., 2025; Rao, Wang, et al., 2025; Razzaq et al., 2022). Similar breeding strategies have been proposed for enhancing crop resilience and nutritional quality (Z. He et al., 2024). Furthermore, the MYB candidates identified here represent promising targets for functional validation and potential CRISPR-mediated modulation to fine-tune phenolic biosynthesis in kiwifruit.

While the present study provides compelling evidence for MYB-linked metabolic divergence, several limitations warrant consideration. The transcriptomic correlations, though statistically robust, remain associated; functional validation through gene overexpression or silencing is necessary to confirm regulatory causality. We did not perform RT-qPCR validation for the identified MYB genes; however, the high reproducibility of our RNA-seq data and strong metabolite correlations support the observed expression patterns. Future studies should include qPCR or CRISPR-based functional assays to validate these MYB regulators. Additionally, metabolomic data is based on relative quantification; absolute quantification of key lignans and coumarins would strengthen the nutritional interpretation. We also acknowledge that a systematic integration of structural enzyme gene expression with metabolite profiles was not performed, as many key enzyme genes in the kiwifruit phenylpropanoid pathway remain uncharacterized and the central goal of this study was to identify MYB regulators. This represents a valuable direction for future research. Future research should integrate enzyme activity assays, promoter analysis, and subcellular localization studies to elucidate the precise regulatory mechanisms. Expanding the analysis to additional *Actinidia* germplasm could further clarify the evolutionary trajectory of phenolic specialization within the genus. While the DPPH assay provides a reliable measure of radical scavenging capacity, it is an in vitro chemical assay. Cellular antioxidant activity assays would offer more physiologically relevant insights, and such analyses are planned for future studies.

## 5 Conclusion

Collectively, this study establishes a link between MYB transcriptional regulation and species-specific phenolic metabolism in *Actinidia* species. The *A. arguta* variety ‘Danyang flat’ exemplifies a lignan/coumarin-dominant metabolic strategy associated with superior radical-scavenging capacity, while *A. chinensis* ‘SunGold’ represents a flavonoid-oriented phenotype. These findings not only deepen our biochemical understanding of phenylpropanoid partitioning but also highlight the potential of underutilized *A. arguta* germplasm as a source of bioactive compounds for functional food development and breeding innovation. Future research should focus on functional validation of candidate MYB regulators, elucidation of downstream enzyme kinetics, and exploration of environmental modulation of phenolic flux. Such integrative approaches will accelerate the design of metabolically informed breeding strategies for high-value kiwifruit cultivars.

## Author contribution

Conceptualization, M.D., S.Y. and M.J.R.; methodology, M.D. and J.C.; software, M.D., S.H. and M.J.R. investigation, M.D., J.C., S.H., Y.L., M.T., C.C., T.L., J.D., S.Y., Q.A., and X.D; writing—original draft preparation, M.D. and M.J.R.; writing—review and editing, All Authors; supervision, M.D., S.Y. and M.J.R.; funding acquisition, M.D., Q.A., and S.Y. All authors have read and agreed to the published version of the manuscript.

## CRediT authorship contribution statement

**Mingzheng Duan:** Writing – review & editing, Writing – original draft, Visualization, Resources, Project administration, Methodology, Investigation, Funding acquisition, Formal analysis, Conceptualization. **Jieyu Chang:** Visualization, Validation, Resources, Methodology, Formal analysis. **Shirong He:** Visualization, Validation, Resources, Methodology, Formal analysis. **Yuanqiao Li:** Visualization, Validation, Investigation, Formal analysis, Data curation. **Congjing Chen:** Visualization, Validation, Software, Investigation, Formal analysis. **Tingfen Lu:** Visualization, Validation, Software, Resources, Investigation, Data curation. **Jinrui Duan:** Writing – review & editing, Visualization, Validation, Software, Resources, Investigation. **Xiande Duan:** Writing – review & editing, Visualization, Validation, Software, Investigation. **Qurban Ali:** Writing – review & editing, Visualization, Validation, Investigation, Funding acquisition, Data curation, Conceptualization. **Shunqiang Yang:** Writing – review & editing, Visualization, Validation, Investigation, Data curation. **Muhammad Junaid Rao:** Writing – original draft, Visualization, Validation, Software, Methodology, Investigation, Formal analysis, Conceptualization.

## Funding information

This work was jointly funded by the United Arab Emirates University for providing a postdoctoral grant on climate action (#12S140); the Project for Reserve Talents of Young and Middle-Aged Academic and Technical Leaders (Grant No. 202305 AC160057); and the Young Talent Project of the Talent Support Program for the Development of Yunnan (Grant No. 210604199008271015).

## Declaration of competing interest

The authors declare that they have no known competing financial interests or personal relationships that could have appeared to influence the work reported in this paper.

## Data Availability

The raw data of RNA-seq are deposited in China National GeneBank (CNGB; https://db.cngb.org/cnsa) under project accession No. CNP0008694.

## Appendix A Supplementary data

Supplementary material 1 Supplementary Fig. S1. Phenol biosynthesis pathway showing key compounds across five kiwifruit varieties. Metabolites are scaled or colored by *Z*-score-normalized abundance per variety.

Supplementary material 2 **Supplementary Table S1**. Comprehensive raw data of all polyphenol metabolites identified across five kiwifruit varieties via widely targeted UPLC-MS/MS analysis. **Supplementary Table S2.** Complete statistical summary of phenolic metabolites across all pairwise varietal comparisons. For each of the ten comparisons among the five kiwifruit varieties (LC, WL, GC, SG, DY), the table lists each identified metabolite alongside its Variable Importance in Projection (VIP) score from OPLS-DA, *p*-value (Student's *t*-test), log₂ fold change (Log2FC), and fold change value. **Supplementary Table S3.** The table lists each of the 40 phenolic compounds designated PP1 through PP40 alongside their full chemical names, as used in hierarchical cluster analysis (HCA) and subsequent statistical comparisons.**Supplementary Table S4.** Transcriptomic dataset from five kiwifruit varieties. The table lists gene expression levels (FPKM and read counts) in biological triplicates, results of differential expression analysis (log₂FC, *p*-value, adjusted *p*-value) for pairwise variety comparisons, and comprehensive functional annotations (KEGG, GO, Pfam, TF family, etc.).
