# Supplementary material for: Integrated widely targeted UPLC-MS/MS metabolomics and transcriptomics reveal MYB-linked variation in bioactive phenolics across five kiwifruit varieties
Source: Food Chem (Oxf). 2026 May 9;12:100410. doi: 10.1016/j.fochms.2026.100410 (PMC13200118; doi:10.1016/j.fochms.2026.100410)
Supplement: Supplementary material 1 — Supplementary Fig. S1. Phenol biosynthesis pathway showing key compounds across five kiwifruit varieties. Metabolites are scaled or colored by Z-score-normalized abundance per variety. [file mmc1.docx]

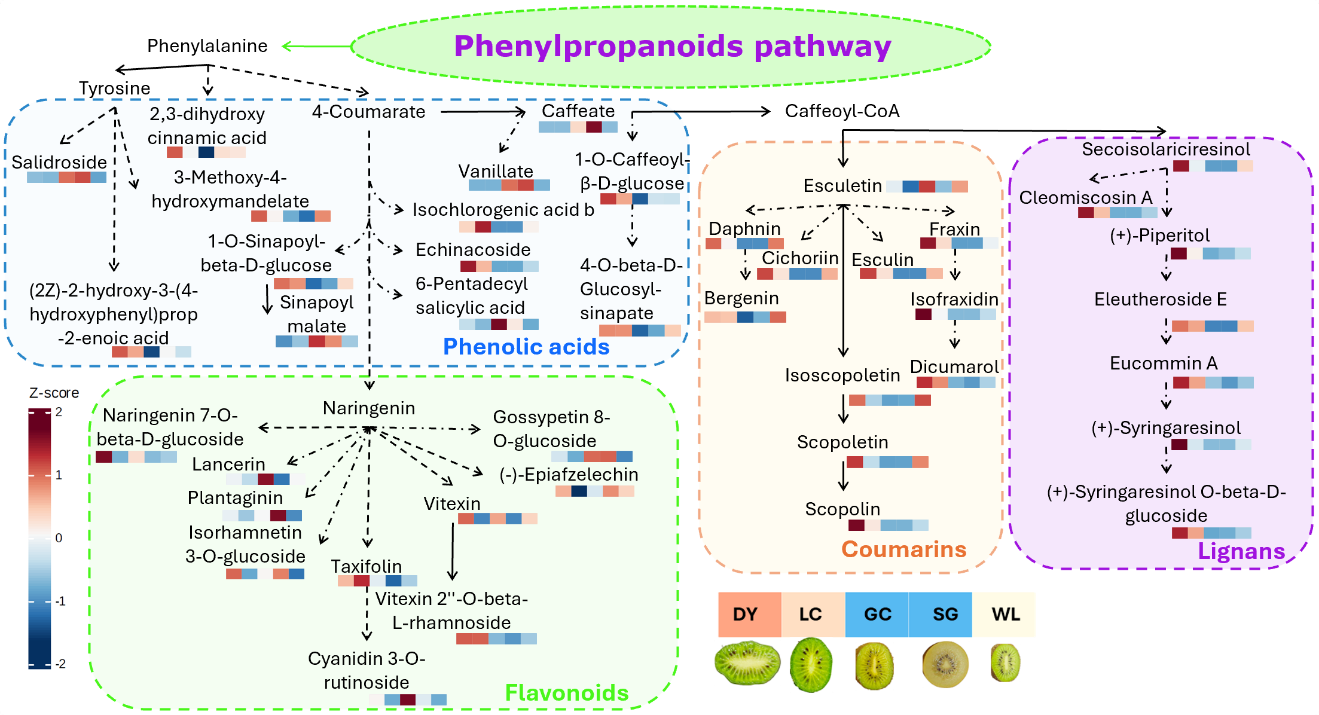


**Supplementary Figure S1.** Phenol biosynthesis pathway showing relative abundances of key compounds across five kiwifruit varieties. Metabolites are scaled or colored by Z‑score‑normalized abundance per variety, emphasizing varietal differences in metabolic flux and end‑product accumulation.
